# Hybridisation Potential of 1',3'-Di-*O*-methylaltropyranoside Nucleic Acids

**DOI:** 10.3390/molecules20034020

**Published:** 2015-03-03

**Authors:** Akkaladevi Venkatesham, Dhuldeo Kachare, Guy Schepers, Jef Rozenski, Mathy Froeyen, Arthur Van Aerschot

**Affiliations:** Medicinal Chemistry, Rega Institute for Medical Research, KU Leuven, Minderbroedersstraat 10, Leuven BE-3000, Belgium; E-Mails: akkaladevi.venkatesham@rega.kuleuven.be (A.V.); ddkachare@gmail.com (D.K.); Guy.Schepers@rega.kuleuven.be (G.S.); Jef.Rozenski@rega.kuleuven.be (J.R.); Mathy.Froeyen@rega.kuleuven.be (M.F.);

**Keywords:** modified oligonucleotides, hexitol nucleic acids, pairing behaviour, hybridisation, constrained oligonucleotides

## Abstract

In further study of our series of six-membered ring-containing nucleic acids, different 1',3'-di-*O*-methyl altropyranoside nucleoside analogs (DMANA) were synthesized comprising all four base moieties, adenine, cytosine, uracil and guanine. Following assembly into oligonucleotides (ONs), their affinity for natural oligonucleotides was evaluated by thermal denaturation of the respective duplexes. Data were compared with results obtained previously for both anhydrohexitol (HNAs) and 3'-*O*-methylated altrohexitol modified ONs (MANAs). We hereby demonstrate that ONs modified with DMANA monomers, unlike some of our previously described analogues with constrained 6-membered hexitol rings, did not improve thermodynamic stability of dsRNA complexes, most probably in view of an energetic penalty when forced in the required 1C4 pairing conformation. Overall, a single incorporation was more or less tolerated or even positive for the adenine congener, but incorporation of a second modification afforded a slight destabilization (except for A), while a fully modified sequence displayed a thermal stability of −0.3 °C per modification. The selectivity of pairing remained very high, and the new modification upon incorporation into a DNA strand, strongly destabilized the corresponding DNA duplexes. Unfortunately, this new modification does not bring any advantage to be further evaluated for antisense or siRNA applications.

## 1. Introduction

Gene silencing has become a standard technique for studying gene functions or in trying to obtain therapeutic effects and theoretically can be attained by interfering with transcription (via formation of triple stranded complexes [1] or translation processes. The latter can be obtained via steric blocking antisense oligonucleotides (ASOs) or via mRNA cleavage of double stranded complexes with RNAseH activating ASOs [2,3,4]. However, the vast majority of researchers nowadays have turned to the use of RNA interference (RNAi)-based strategies, which has recently become the technique of choice to silence gene expression in mammalian cell culture and is envisaged as a first choice for therapeutic treatment as well [5,6,7]. However, we need to point out that at the moment only one aptamer [8] and one antisense oligonucleotide have been effectively FDA approved for gene silencing [9], and also exon-skipping oligonucleotides are receiving considerable attention [10].

In cell culture in general unmodified siRNAs are highly efficient, however for *in vivo* application some chemical modifications are warranted to stabilise the siRNAs and to increase their selectivity and to promote delivery [11,12]. In the past, we and others have studied a wide variety of strategies for both ASO and siRNA modification as reviewed several times [13,14,15].

Our group has been very successful in increasing the affinity for RNA using modified building blocks based on 6-membered hexitol rings which resulted in the hexitol nucleic acids series [16,17,18,19]. However, the LNA monomers of the Wengel group [20] consistently showed the strongest affinity for RNA, and the series comprises many alternative structures [21]. Overall, both our hexitol nucleic acids and the LNA series of compounds take on a pre-organized conformation, fitting the A-form of dsRNA and rationalizing the strong hybridization characteristics noticed.

**Figure 1:**
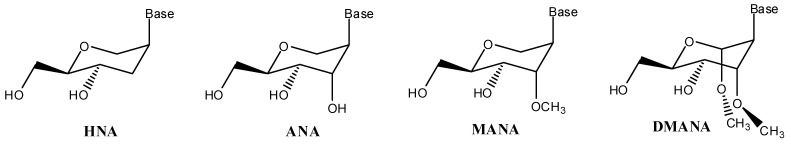
Structures of the different hexitol based nucleic acids (HNA, ANA and MANA) as discussed above and the newly envisaged structure DMANA, based on methyl-altropyranoside.

Herein, hexitol nucleic acids (HNA, Figure 1) are composed of 2,3-dideoxy-d-*arabino*-hexitol units with a nucleobase situated in the 2-(*S*)-position (in *cis* to the hydroxymethyl substituent as in natural nucleosides). Addition of a supplementary hydroxyl at the 3'-α-position resulted in d-altritol nucleic acid (ANA, Figure 1) analogs with increased affinity for RNA strands [18,22]. More recently, we finally reported on the 3'-*O*-methylated ANA congeners (MANA, Figure 1) resulting in a further increase of 0.5 °C/modification when evaluating melting temperatures (T_m_) upon hybridisation to RNA [23]. Herein, both the heterocyclic base and the 3'-*O*-methyl moiety are located in an axial position with a ^4^C_1_ conformation. However, assembly of the hexitol series of nucleosides is long-routed starting with synthesis of the 1,5-anhydrohexitol ring. In view of the positive results obtained for the MANA series of congeners and the abundance of cheap α-d-methylglucoside, we now planned to prepare and evaluate “bis-methylated altritol nucleosides”, or more correctly di-*O*-methylated altropyranoside nucleic acids (DMANA, Figure 1).

## 2. Results and Discussion

### 2.1. Chemical Synthesis of the New Building Blocks

**Scheme 1:**
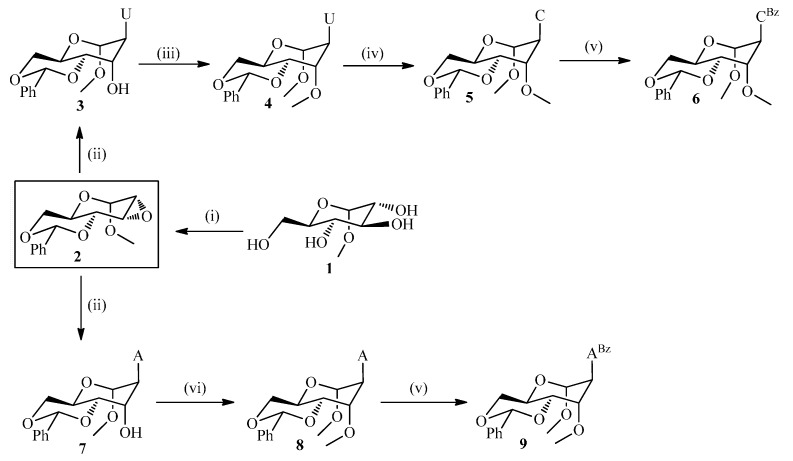
Synthetic scheme of the protected DMANA congeners for uracil, cytosine and adenine.

The 1'-*O*-methylglycosidic protected analogues **4**, **6** and **9** (Scheme 1) were obtained starting from ubiquitous methyl glucopyranoside **1**, which in three steps was converted to **2** in 51.4% overall yield according to literature procedures [24]. Herein, regioselective epoxide ring opening of **2** with the sodium salts of uracil or adenine in DMF at 120–130 °C afforded the corresponding altrohexitol derivatives **3** and **7** in 66%–85% yield. Chemoselective methylation of **7** and **3** was accomplished using NaH, MeI in dry THF at low temperature for 1 h to afford the methylated nucleosides **4** and **8** in 65% and 75% yield, respectively. The selective *O*- *vs*. *N*-methylation mainly depends upon the dielectric constant of the solvent [23,25] and the stability of sodium-enolate chelation [26]. Hence, low dielectric constant and high chelation stabilizing capacity of THF afforded a higher *O*-selectivity compared to DMF. The one pot conversion of compound **3** to the triazolide derivative [27] using 1,2,4-triazole, POCl_3_, and triethylamine, and subsequent treatment with aqueous ammonia/dioxane (1:1) at ambient temperature for 18 h yielded the cytidine derivative (**5**) with 58% (overall yield for 2 steps). Base protection of **5** and **8** using benzoylchloride in pyridine at rt for 3 h afforded **6** and **9** in 83 and 88% yield, respectively.

**Scheme 2:**
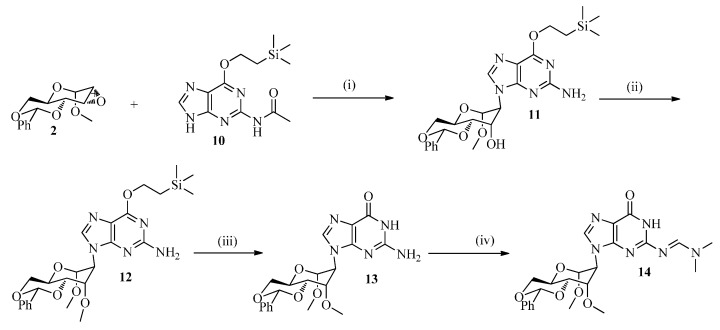
Synthetic scheme for the protected guanine containing analog.

Due to the scalability, solubility and reproducibility of the reaction it proved advantageous to use the guanine derivative **10** for the epoxide ring opening reaction. The latter was obtained via Mitsunobu reaction of *N*^2^-acetylguanine [28] and 2-(trimethylsilyl)ethanol in analogy with the previously described protocol for *O*^6^-[2-(*p*-nitrophenyl)ethyl]guanine [29]. Selective epoxide ring opening was accomplished with the lithium salt of **10** (Scheme 2) in DMF at 130 °C and afforded 41% of **11** along with 26% of recovered **10**. Remarkably, the acetyl protection was lost in **11** upon the prolonged heating in DMF. Further chemoselective methylation using NaH (60%) and MeI in DMF and DCM at low temperature for 4 h gave **12** in 94% yield. Deprotection of **12** was done by 1 M TBAF in THF at rt for 2 h to yield 75% of **13**. The more base labile *N*^2^-dimethylformamidine (dmf) [30,31,32] group was introduced using *N*,*N*-dimethylformamide diethylacetal in methanol under reflux for 12 h to afford **14** with 85% yield.

*Reagents and Conditions*: (i) (a) C_6_H_5_CHO, ZnCl_2_, 72 h (66%); (b) 6 eq. CH_3_C_6_H_4_SO_2_Cl, pyridine, 60 °C, 72 h (78%); (c) 5.3 M in MeOH, CH_2_Cl_2_, rt, 12 h (99%); (ii) NaH (60%), DMF, 20 °C, 12 h (33.3% of **3** along with 40.1% recovery of **2** and 75% of 7); (iii) NaH (60%), CH_3_I, THF, 0 °C, 1 h (64.7%); (iv) (a) POCl_3_, 1*H*-1,2,4-triazole, Et_3_N, pyridine, rt, 2 h; (b) 1,4 dioxane, aq. NH_3_, rt, 12 h (58.17% overall 2 steps); (v) Benzoyl chloride, pyridine, rt, 3 h (87.6% of **6** and 83% of **9**; (vi) NaH (60%), CH_3_I, THF, −78 °C, 4.5 h to −30 °C, 1 h (75%).

*Reagents and Conditions*: (i) LiH, DMF, 130 °C, 18 h (42% of **11** along with 26% recovery of **10**); (ii) NaH (60%), CH_3_I, DMF, DCM, −30 °C to −20 °C, 4 h (94%); (iii) TBAF, THF, rt, 2 h (75%); (iv) Me_2_NCH(OEt)_2_, MeOH, reflux for 12 h (85%).

**Scheme 3:**
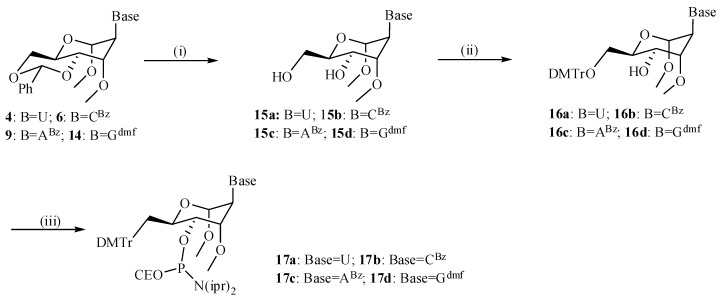
Scheme for assembly of the different phosporamidites.

Deprotection of the benzylidene protecting group under mild conditions using AcOH:H_2_O (3:1) at 45 °C for 12 h afforded **15a**–**d** in 50%–94% yield (Scheme 3), which was followed by classical dimethoxytritylation and phosphitylation. Hereto, the nucleosides **15a**–**d** were selectively protected at the 6'-OH by reaction with DMTrCl in pyridine at rt for 3 h to furnish the corresponding protected derivatives **16a**–**d** in 74%–93% yield. Phosphitylation of **16a**–**d** at the 4'-OH with 2-cyanoethyl *N*,*N*-diisopropylchlorophosphoramidite in anhydrous CH_2_Cl_2_ at 0 °C for 1.5 h afforded the corresponding phosphoramidite building blocks **17a**–**d** in 65%–93% yield, to be used for oligomer assembly. Assembly of all oligonucleotides and purification was carried out as described before [33].

*Reagents and Conditions*: (i) AcOH: H_2_O (3:1), 45 °C, 12 h (50% to 94%); (ii) DMTrCl, pyridine, rt, 3 h (74% to 93%); (iii) (iPr)_2_N(OCE)PCl, DIPEA, DCM, 0 °C, 1.5 h (65% to 93%).

### 2.2. Oligonucleotide Affinity Measurements

**Figure 2:**
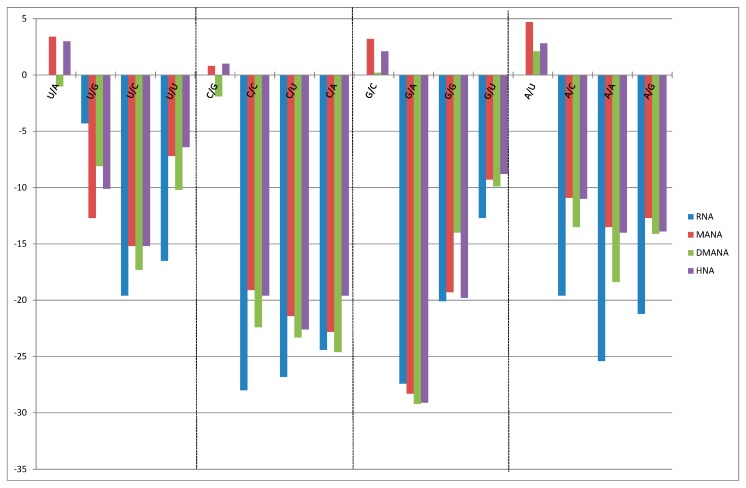
Base pairing selectivity for different 6-membered ring analogues. Conditions: graphical overview of the (de)stabilization of the matched and mismatched nonamer sequences following a single incorporation of a sugar modified nucleoside (MANA in red, DMANA in green and HNA in purple) wherein the selectivity of pairing is shown by destabilization of the respective mismatches. The pairing selectivity for RNA is shown in blue.

First, following incorporation of a single DMANA modification into an dsRNA nonamer sequence [5'-GCGU-X*-UGCG/5'-CGCAYACGC], the respective affinities for complementary RNA were studied in a 0.1 M NaCl buffer and were compared with the melting temperatures (T_m_) of previously studied six-membered ring structures substituting for the ribose ring (Table 1). Within the context of all four natural bases, the HNA (anhydrohexitol) and ANA (altritol) substitutions proved advantageous and considerably stabilized the RNA helix. Where ANA modifications were either slightly less stabilizing (for pyrimidines) or more stabilizing (as with purines) *versus* HNA modifications, methylation of the 3'-hydroxyl moiety of ANA congeners further improved the affinity for RNA systematically with approximately 0.5 °C. However, converting the hexitol into a methyl hexopyranoside via attachment of a second “methoxy substituent” at the 1'-position as in our DMANA constructs, wiped out the advantage which was gained before in using constrained hexitol moieties. The obtained affinities of these DMANA containing constructs more or less matched those of the corresponding fully complementary RNA sequences. Incorporation of a single DMANA building block slightly destabilized the RNA duplex for substitution of a pyrimidine, but on contrast slightly stabilized the duplex in case of guanine and to a larger extent upon substitution of adenine within this sequence context (Table 1 and Figure 2, base matches).

As on average the DMNA pairing affinity to RNA was not advantageous nor really detrimental, we further studied their mismatch behavior within the same RNA nonamer constructs (5'-GCGU-X*-UGCG/5'-CGCAYACGC; Figure 2) in comparison to different constructs. The graphical chart displays the destabilization in relation to the respective matched sequence as obtained for DMANA building blocks in comparison to discrimination properties for HNA and MANA ([23] and within dsRNA duplexes. Analogous discrimination properties were noted, and especially for dmanaC very selective pairing to guanosine was obtained with −22 °C to −24 °C of destabilization for the different mismatches. However, no overall advantage can be seen in terms of either pairing selectivity or universal base pairing capabilities. Hence, normal WC pairing can be assumed.

**Table 1:** T_m_ values of complementary RNA duplexes [5'-GCGU-X*-UGCG/5'-CGCAYACGC].

X*	Structure	T_m_ (°C)	X*	Structure	T_m_ (°C)
U*	RNA	50.4 ± 0.0	G*	RNA	60.4 ± 0.0
	HNA	53.4 ± 0.1		HNA	62.4 ± 0.1
	ANA	53.0 ± 0.2		ANA	62.9 ± 0.1
	MANA	53.8 ± 0.2		MANA	63.4 ± 0.1
	DMANA	50.6 ± 0.2		DMANA	61.6 ± 0.1
C*	RNA	60.8 ± 0.1	A*	RNA	52.5 ± 0.1
	HNA	62.0 ± 0.0		HNA	55.0 ± 0.2
	ANA	60.9 ± 0.1		ANA	56.5 ± 0.1
	MANA	61.4 ± 0.0		MANA	57.0 ± 0.1
	DMANA	60.1 ± 0.2		DMANA	55.5 ± 0.1

Conditions: as determined in 100 mM NaCl buffer containing 20 mM KH_2_PO_4_ and 0.1 mM EDTA, pH 7.5, with a duplex concentration of 4 µM. Annotations U*, C*, A* and G* denote either a RNA, HNA, ANA, MANA or DMANA monomer respectively, *versus* the natural complementary base Y.

This picture was confirmed with a second substitution within different nonamer dsRNA sequences (Table 2), with the largest destabilization noted for dmanaC building blocks (−1.8 °C/modification) while still an increase in stability of 1.1 °C/modification was seen with incorporation of 2 dmanaA blocks. The stabilizing effects of the HNA and 3'-methylated ANA constructs (MANA) are included for comparative reasons. We therefore decided to prepare a fully modified DMANA octamer and hybridized it to the complementary RNA sequence (Table 2, bottom). Where the natural RNA duplex displayed a Tm of 40.6 °C, the DMANA construct still paired albeit with slightly lower affinity (−0.3 °C/modification). The different hexitol constructs on the other hand strongly increased the affinity for the RNA complement as documented before [23,34]. It hence can be concluded that the DMANA analogues are different from the previous hexitol series of compounds with a constrained 6-membered ring conformation fit for pairing to RNA.

**Table 2:** Thermal stability for RNA duplexes containing a double modification.

Sequences	X*	Tm (°C)	∆Tm/Modification (°C)
5'-GCU*GUGU*CG-3'	RNA	55.6 ± 0.4	Reference
	MANA	62.6 ± 0.2	3.5
	DMANA	54.5 ± 0.1	−0.5
	HNA	60.2 ± 0.1	2.3
5'-GCC*AUAC*CG-3'	RNA	57.1 ± 0.1	Reference
	MANA	59.3 ± 0.1	1.1
	DMANA	53.4 ± 0.1	−1.8
	HNA	58.2 ± 0.1	0.6
5'-GCG*UUUG*CG-3'	RNA	51.3 ± 0.2	Reference
	MANA	54.3 ± 0.1	1.5
	DMANA	51.1 ± 0.1	−0.1
	HNA	53.0 ± 0.2	0.8
5'-GCA*CUCA*CG-3'	RNA	57.1 ± 0.1	Reference
	MANA	63.5 ± 0.1	3.2
	DMANA	59.3 ± 0.1	1.1
	HNA	62.1 ± 0.1	2.5
5'-G*C*G*U*A*G*C*G*-3'	RNA	40.6 ± 0.1	Reference
	MANA	61.1 ± 0.3	2.6
	DMANA	38.1 ± 0.2	−0.3
	ANA	59.6 [34]	2.4
	HNA	52.0 [34]	1.4

Conditions: T_m_ as determined in 100 mM NaCl buffer containing 20 mM KH_2_PO_4_ and 0.1 mM EDTA, pH 7.5, with a duplex concentration of 4 µM. U*, C*, A* and G* denote either a RNA, MANA, DMANA, ANA or a HNA monomer, respectively. At the bottom the results are given for the fully modified strands paired to the complementary RNA strand.

Finally, incorporation of a single DMANA building block into dsDNA 13-mer sequences [5'-CACCGX*TGCTACC-3'/3'-GTGGCYACGATGG-5'] was evaluated at 0.1 M salt concentration (Table 3) for both match and mismatch sequences. However, a single DMANA incorporation already afforded respectively 9 °C (for dmanaU and dmanaC), 5 °C (for dmanaA) or 6 °C (for dmanaG) of destabilization for the different matched pairs within this sequence context. A slightly better result could be expected if we could compare the dmanaT construct instead of dmanaU in view of the stabilizing effect of a 5-methyl substituent on the base, but this still would have resulted in a destabilization of 7 to 8 °C. The selectivity of pairing is adequate but gives a mixed picture, with selectivity being dependent probably on the base and the sequence context. In view of the fairly strong destabilization *versus* DNA sequences it is clear however that these DMANA blocks do not have a DNA like conformation.

**Table 3:** Hybridization studies following incorporation into a DNA strand.

Y	A		T		G		C	
X* (X)	T_m_	ΔT_m_	T_m_	ΔT_m_	T_m_	ΔT_m_	T_m_	ΔT_m_
U*	47.7	-	40.9	−6.8	41.7	−6.0	38.3	−9.4
T	57.1	-	46.7	−10.4	50.3	−6.8	44.2	−12.9
A*	41.9	−10.8	52.7	-	45.3	−7.4	41.4	−11.3
A	46.6	−10.7	57.3	-	52.9	−4.4	44.4	−12.9
C*	41.8	−10.3	40.8	−11.3	52.1	-	38.5	−13.6
C	44.4	−16.5	45.6	−15.3	60.9	-	40.6	−20.3
G*	42.9	−11.0	48.9	−5.0	46.0	−7.9	53.9	-
G	51.6	−8.4	51.0	−9.0	53.5	−6.5	60.0	-

Conditions: T_m_ values are provided for a single incorporation of a DMANA (X*) modification into a mixed DNA sequence [5'-CACCGX*TGCTACC-3'/3'-GTGGCYACGATGG-5'] for the match and the different mismatch sequences (Y) at 0.1 M salt and 4 M of duplex concentration.

### 2.3. Discussion

The MedChem group of the Rega Institute has already been elaborating for many years on nucleoside analogues with a 6-membered ring system substituting for ribose for various applications. Especially the analogues with a 1,5-anhydrohexitol ring having the base at the C2' in syn orientation with the remaining hydroxymethyl substituent (HNA, ANA and MANA) turned out to be well pre-organized for pairing with RNA, and are thus strongly stabilizing for RNA duplexes. Several biological studies have been undertaken with both HNA and ANA [22,35,36] and interesting results were obtained more recently regarding their use as xeno-nucleic acids [37,38]. Highest affinities so far however were obtained with MANA building blocks [23]. We therefore started to study the influence of DMANA analogues carrying an additional methoxy substituent on the 6-membered ring system. However, as shown by the various T_m_ studies, incorporation of the new modification does not further increase the affinity for RNA, but overall rather tends to slightly destabilize dsRNA complexes, while strongly destabilizing DNA upon incorporation. Therefore the modification still resembles more closely RNA monomers with their 3'-endo conformation as in RNA duplexes.

**Figure 3:**
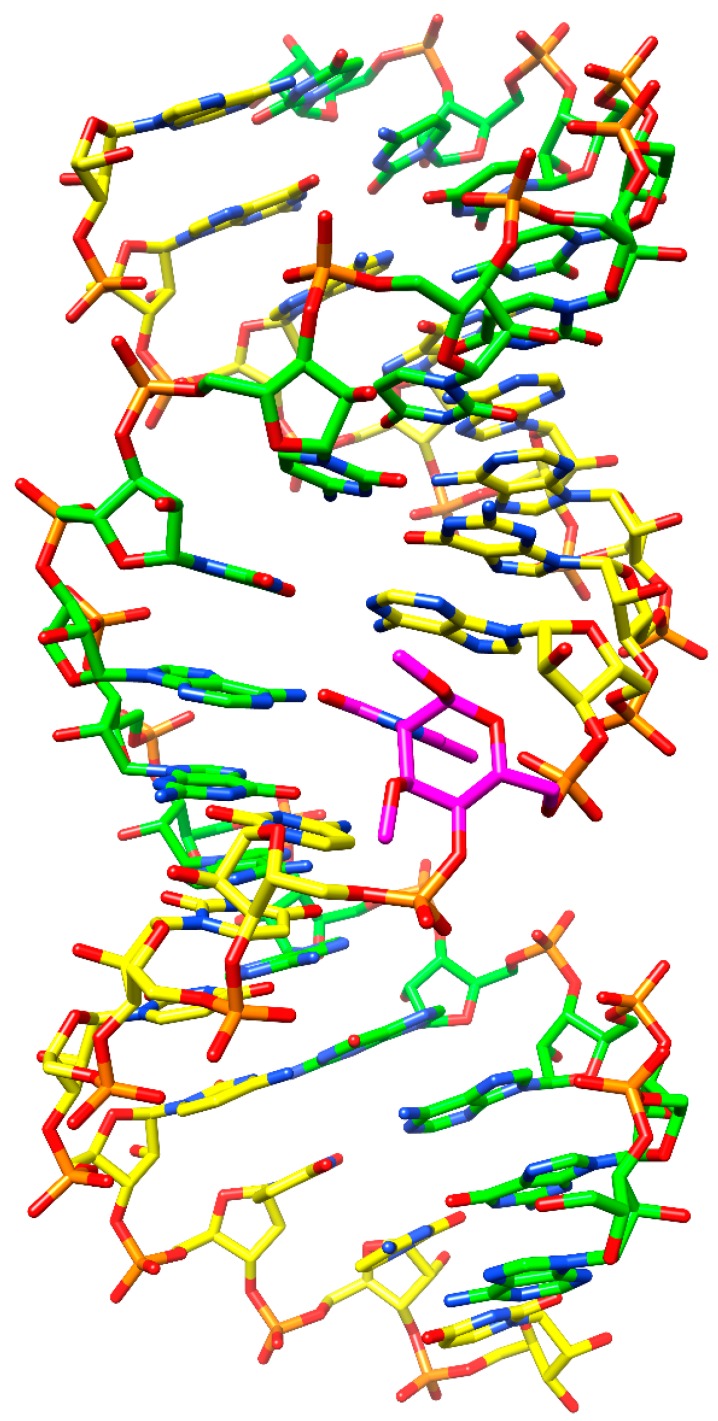
RNA duplex following insertion of a DMANA modification (DMANA modification in purple; picture generated using Chimera (UCSF Chimera—a visualization system for exploratory research and analysis [41]).

These findings are further corroborated by inserting *in silico* a modified building block into a dsRNA. As can be seen in Figure 3, no steric hindrance occurs when substituting a DMANA residue having two OMe groups at the sugar 1' and 3' positions for a uridine into an RNA duplex for which the model of Mooers was used [39]. The HNA sugar conformation upon pairing with RNA is ^1^C_4_ having an axial base orientation. Likewise, for the DMANA structure incorporated into the dsRNA, both methoxy substituents likewise are oriented axially with no apparent steric clashes. However, it is well known that apart from forces like the anomeric effect, substituents in 6-membered rings prefer the equatorial orientation to avoid 1,3-diaxial interactions. Energy calculations for the monomers using Amber force field [40] indeed show a slight preference for having the base and both methoxy groups in an all-equatorial configuration (with 4'-OH and 6'-CH_2_OH in axial orientation), opposite to what is found for HNA and ANA building blocks with an axial oriented heterocyclic base. Increasing the number of OMe substituents therefore may destabilize the ^1^C_4_ chair conformation giving preference to ^4^C_1_, which is less compatible with the RNA duplex. Hence, the energy penalty to preserve the DMANA monomer in a ^1^C_4_ conformation to allow for pairing within a dsRNA strand, might upset the entropic gain as expected of a pre-organized monomer. 

Table 4 indeed shows the largest energy difference for both chair conformations for DMANA constructs. This energetic penalty for a forced change in conformation could be the basis of the reduced fitness of DMANA analogues for pairing with RNA.

**Table 4:** Energy calculations for monomers with different scaffold.

Base and OMe Orientation	Axial	Equatorial	Base and OMe Orientation	Axial	Equatorial
HNA	−205.31	−205.79	MANA	−195.22	−200.35
ANA	−200.58	−198.19	DMANA	−187.62	−194.82
Chair	^1^C_4_	^4^C_1_	Chair	^1^C_4_	^4^C_1_

Conditions: Final energies in kcal/mol are shown following minimization using Amber force field. Nucleotides all have a thymine base, uncharged residues (phosphate groups protonated), with parametrization via antechamber, Gaff force field, 5000 steps of energy minimization, born solvation energy model.

## 3. Experimental Section

### 3.1. General

All chemicals including methylglucopyranoside were provided by Sigma-Aldrich (Diegem, Belgium) or Acros Organics (Geel, Belgium) and were of the highest quality. ^1^H and ^13^C-NMR spectra were determined with a 300, 500 and 600 MHz Varian Gemini apparatus (currently Agilent Technologies, Santa Clara, CA, USA) with tetramethylsilane as internal standard for the ^1^H NMR spectra (s = singlet, d = doublet, dd = double doublet, t = triplet, br. s = broad signal, m = multiplet) and the solvent signal; CD_3_OD-*d*_4_ (δ = 48.9 ppm), DMSO-*d*_6_ (δ = 39.6 ppm) or CDCl_3_ (δ = 76.9 ppm) for the ^13^C-NMR spectra. Exact mass measurements were performed with a quadrupole/orthogonal acceleration time-of-flight tandem mass spectrometer (qTOF2, Micromass, Manchester, UK) fitted with a standard electrospray ionization (ESI) interface. All solvents were carefully dried or bought as such.

### 3.2. 1',5';2',3'-Dianhydro-4',6'-O-benzylidene-1'-O-methyl-d-allopyranoside (**2**)

To a solution of the 4',6'-*O*-benzylidene-2',3'-ditosyl intermediate (10.0 g, 17 mmol) in CH_2_Cl_2_ (80 mL) was added NaOMe (5.3 M in MeOH, 12.8 mL, 67.8 mmol). After stirring for overnight at room temperature, the reaction mixture was concentrated and the residue was dissolved in CH_2_Cl_2_ (150 mL), and washed twice with brine (50 mL). The aqueous layer was again extracted with CH_2_Cl_2_ (2 × 80 mL). Combined organic layers were dried over anhydrous Na_2_SO_4_*,* filtered and evaporated to get pure epoxide **2** (4.45 g, 99%). ^1^H-NMR (300 MHz, CDCl_3_): δ 7.60–7.33 (m, 5H, Ar-H), 5.60 (d, *J* = 6.1 Hz, 1H, Ph-CH), 4.92 (dd, *J* = 6.1, 2.7 Hz, 1H, 1'-H), 4.32–4.22 (m, 1H, 6'-He), 4.18–4.05 (m, 1H, 5-H), 3.98 (dd, *J* = 9.0, 6.2 Hz, 1H, 4-H), 3.71 (m, 1H, 6-Ha), 3.58–3.45 (m, 5H, 2'-H, O-Me). ^13^C-NMR (75 MHz, CDCl_3_): δ 137.11 (Ar-C_i_); 129.19; 128.28 (Ar-C_p+o_); 126.27 (Ar-C_m_); 102.71 (Ph-C); 95.27 (C-1'); 77.83 (C-4'); 68.86 (C-6'); 59.99 (C-5'); 55.82 (OMe); 53.07 (C-3'); 50.66 (C-2'); HRMS calcd. for C_14_H_16_O_5_Na^+^ [M+Na]^+^ 287.0890, found 287.0891.

### 3.3. 4',6'-O-Benzylidene-1'-O-methyl-2'-deoxy-2'-(uracil-l-yl)-d-altropyranoside (**3**)

To a solution of uracil (4.62 g, 38.6 mmol) in dry DMF (30 mL) was added NaH (60% dispersion in oil, 1.29 g, 41.2 mmol). The reaction mixture was heated at 120 °C under an argon atmosphere for 1 h and to this reaction mixture epoxide **2** (3.4 g, 12.9 mmol) in dry DMF (20 mL) was added and stirring was continued for overnight at same temperature. The reaction mixture was then cooled and evaporated to dryness. The residue was dissolved in ethyl acetate (150 mL) and the organic layer was washed with a saturated aqueous NaHCO_3_ solution (2 × 50 mL). The aqueous layer was again extracted with EtOAc (3 × 50 mL). The combined organic layers were washed with brine (2 × 50 mL), dried over Na_2_SO_4_, and concentrated under *vacuo* and purification by silica gel column chromatography (elution with 2% MeOH in DCM) afforded **3** (1.61 g, 33%) as a white foam while recovering a large part of the starting epoxide **2** (1.94 g, 40% yield). ^1^H-NMR (500 MHz, CDCl_3_): δ 7.75 (d, *J* = 8.1 Hz, 1H, 6-H), 7.49–7.41 (m, 2H, Ar-H), 7.39–7.30 (m, 3H, Ar-H), 5.77 (d, *J* = 8.1 Hz, 1H, 5-H), 5.62 (s, 1H, Ph-CH), 4.83 (s, 1H, 1'-H), 4.81 (s, 1H, 2'-H), 4.48 – 4.38 (m, 2H, 5'-H, 6'-He), 4.16 (brs, 1H, 3'-H), 3.81 (t, *J* = 9.9 Hz, 1H, 6'-Ha), 3.69 (dd, *J* = 9.9, 2.4 Hz, 1H, 4'-H), 3.41–3.51 (m, 4H, OMe, -OH). ^13^C-NMR (125 MHz, CDCl_3_): δ 163.08 (C-4); 150.60 (C-2); 141.14 (C-6); 136.81 (Ar-C_i_); 129.26 (Ar-C_p_); 128.28 (Ar-C_m_); 126.18 (Ar-C_o_); 102.91 (C-5); 102.27 (Ph-C); 98.99 (C-1'); 75.61 (C-3'); 68.94 (C-4'); 67.02 (C-6'); 58.19 (C-5'); 57.91 (OMe); 55.97(C-2'). HRMS calcd. for C_18_H_20_N_2_O_7_Na^+^ [M+Na]^+^ 399.1163, found 399.1156.

### 3.4. 4',6'-O-Benzylidene-1',3'-di-O-methyl-2'-deoxy-2'-(uracil-l-yl)-d-altropyranoside (**4**)

To a solution of **3** (1.51 g, 4.0 mmol) in dry THF (15 mL) was added NaH (60% dispersion in oil, 404 mg, 12.04 mmol) at 0 °C and the reaction mixture was stirred at 0 °C for 1h under argon atmosphere. Methyl iodide (0.37 mL, 6.02 mmol) in dry THF (1 mL) was added and stirring was continued for another 1 h at the same temperature. The reaction mixture was quenched with 5 mL MeOH. The solution was concentrated and dissolved in ethyl acetate (150 mL) and washed with saturated aqueous NaHCO_3_ (150 mL). The aqueous layer was again extracted with ethyl acetate (3 × 50 mL). The combined the organic layers were dried over Na_2_SO_4_, filtered, concentrated under *vacuo*, and purification by silica gel column chromatography (elution with 2% MeOH in DCM) afforded the dimethylated nucleoside **4** (1.01 g, 65%). ^1^H-NMR (500 MHz, CDCl_3_): δ 9.41 (s, 1H, N^3^-H), 7.80 (d, *J* = 8.2 Hz, 1H, 6-H), 7.47–7.35 (m, 5H, Ar-H), 5.80 (dd, *J* = 8.1, 1.7 Hz, 1H, 5-H), 5.55 (s, 1H, Ph-CH), 4.90 (d, *J* = 1.7 Hz, 1H, 1'-H), 4.80 (s, 1H, 2'-H), 4.48–4.36 (m, 2H, 6'-He, 3'-H), 3.77 (t, *J* = 10.3 Hz, 1H, 6'-Ha),3.72–3.67 (m, 2H, 4'-H, 5'-H), 3.62 (s, 3H, OMe), 3.46 (s, 3H, OMe). ^13^C-NMR (125 MHz, CDCl_3_): δ 162.98 (C-4); 150.39 (C-2); 141.18 (C-6); 137.06 (Ar-C_i_); 129.20 (Ar-C_p_); 128.28 (Ar-C_m_); 126.21 (Ar-C_o_); 102.86 (C-5); 102.48 (Ph-C); 98.84 (C-1'); 76.06 (C-3'); 75.95 (C-4'); 69.11 (C-5'); 59.59 (C-6'); 58.56 (OMe); 55.94 (OMe); 55.81(C-2'). HRMS calcd. for C_19_H_23_N_2_O_7_ [M+H]^+^ 391.1500, found 391.1494.

### 3.5. 4',6'-O-Benzylidene-1',3'-di-O-methyl-2-deoxy-2'-(cytosin-1-yl)-d-altropyranoside (**5**)

A solution of triazole (1.05 g, 15.2 mmol) and phosphorus oxychloride (0.3 mL, 3.18 mmol) was prepared in pyridine (8 mL) at 0 °C. Triethylamine (2.03 mL, 14.5 mmol) was added dropwise at 0 °C and the solution was stirred 30 min. The uracil derivative **4** (0.650 g, 1.66 mmol) dissolved in dry pyridine (8 mL) was added at 0 °C and the solution was stirred for 2 h at room temperature and concentrated and co-evaporated with toluene (2 × 20 mL). The crude product was dissolved with DCM (150 mL) and washed twice with brine (60 mL). The aqueous layer was extracted with DCM (30 mL). Combined organic layers were dried over anhydrous Na_2_SO_4_, filtered and evaporated. The residue was dissolved in 1,4-dioxane (30 mL), cooled to 0 °C and aqueous ammonia 25% (13 mL) were added. The solution was left overnight at RT. The solution was evaporated and co-evaporated with toluene (3 × 20 mL). The residue was purified by silica gel column chromatography (elution with 10% MeOH/dichloromethane) and afforded the dimethylated cytidine analog **5** (377 mg, 58%) as a white foam. ^1^H-NMR (500 MHz, CD_3_OD): δ 7.91 (d, *J* = 7.5 Hz, 1H, 6-H), 7.50–7.42 (m, 2H, Ar-H), 7.38–7.26 (m, 3H, Ar-H), 5.95 (d, *J* = 7.5 Hz, 1H, 5-H), 5.63 (s, 1H, Ph-CH), 4.93 (s, 1H, 2'-H), 4.91 (d, *J* = 2.1 Hz, 1H, 1'-H), 4.34–4.30 (m, 1H, 6'-He), 4.28 (dd, *J* = 9.9, 5.3 Hz, 1H, 5'-H), 3.84 (dd, *J* = 11.0, 2.3 Hz, 1H, 6'-Ha), 3.81 (dd, *J* = 6.6, 2.3 Hz, 1H, 4'-H), 3.67 (t, *J* = 2.3 Hz, 1H, 3'-H), 3.57 (s, 3H, OMe), 3.42 (s, 3H, OMe). ^13^C-NMR (125 MHz, CD_3_OD): δ 167.49 (C-4); 158.15 (C-2); 144.10 (C-6), 139.09 (Ar-C_i_), 129.97 (Ar-C_p_), 129.07 (Ar-C_m_), 127.44 (Ar-C_o_), 103.41 (Ph-C), 100.28 (C-5), 96.46 (C-1'), 77.40 (C-3'), 76.86 (C-4'), 70.05 (C-6'), 59.93 (C-5'), 59.13 (OMe_)_, 57.45 (OMe), 55.84 (C-2'). HRMS calcd. for C_19_H_24_N_3_O_6_^+^ [M+H]^+^ 390.1654, found 390.1653.

### 3.6. 4',6'-O-Benzylidene-1',3'-O-methyl-2'-deoxy-2'-(N^6^-benzoylcytosin-1-yl)-d-altropyranoside (**6**)

The analog **5** (0.35 g, 0.9 mmol) was co-evaporated with dry pyridine (6 mL), dissolved in dry pyridine (4 mL), and cooled at 0 °C. Benzoyl chloride (0.314 mL, 2.7 mmol) was added and the solution was allowed to come to RT. The solution was stirred for 3 h at RT. The mixture was cooled to 0 °C and water (0.25 mL) was added. Then, aqueous ammonia 25% (2 mL) was added and the solution was stirred for 30 min at RT. The volatiles were removed under reduced pressure and co-evaporated, each time with toluene (3 × 5 mL). The residue was adsorbed on silica by co-evaporation from DCM and purified by silica column chromatography (elution with 2% MeOH/DCM) affording the protected nucleoside **6** (370 mg, 83% yield) as a white foam. ^1^H-NMR (300 MHz, CDCl_3_): δ 8.24 (d, *J* = 7.5 Hz, 1H, 6-H), 7.94 (d, *J* = 7.3 Hz, 2H, Bz-H), 7.70–7.33 (m, 9H, 5-H, Ar-H, Bz-H), 5.56 (s, 1H, Ph-CH), 5.12 (d, *J* = 1.6 Hz, 1H, 1'-H), 4.90 (s, 1H, 2'-H), 4.55–4.37 (m, 2H, 5'-H, 6'-He), 3.86–3.71 (m, 2H, 6'-Ha, 3'-H), 3.74 (dd, *J* = 9.5, 3.1 Hz, 1H, 4'-H), 3.68 (s, 3H, OMe), 3.49 (s, 3H, OMe). ^13^C-NMR (75 MHz, CDCl_3_): δ 167.76 (PhCONH); 162.14 (C-4); 137.11 (C-2); 133.34 (C-6); 129.18, 129.08, 128.28, 127.60, 126.23 (Ar-C, Bz-C); 102.48 (Ph-C); 99.00 (C-5); 97.14 (C-1'), 78.35 (C-3'), 75.77 (C-4'); 75.33 (C-5'); 69.20 (C-6'); 59.51 (OMe); 58.72 (OMe); 56.03 (C2'). HRMS calcd. for C_26_H_28_N_3_O_7_^+^ [M+H]^+^ 494.1922, found 494.1919.

### 3.7. 4',6'-O-Benzylidene-1'-O-methyl-2'-deoxy-2'-(adenin-9-yl)-d-altropyranoside (**7**)

To a solution of adenine (3.22 g, 23.85 mmol) in dry DMF (40 mL) was added NaH (60% dispersion in oil, 890 mg, 22.26 mmol). The reaction mixture was heated at 90 °C under argon atmosphere for 1 h, after which the epoxide **2** (2.1 g, 7.95 mmol) in dry DMF (15 mL) was added and stirring was continued overnight at 120 °C. The reaction mixture was quenched with MeOH (15mL) and following evaporation the residue was partitioned between EtOAc and saturated aqueous NaHCO_3_ solution. The organic layer was separated and the aqueous layer was extracted with EtOAc (3 × 50 mL). The combined the organic layers were dried over Na_2_SO_4_, filtered, concentrated *in vacuo*, and purification by normal silica gel column chromatography (elution with 3% MeOH in DCM) afforded compound **7** (2.37 g, 75%). ^1^H-NMR (300 MHz, CDCl_3_): δ 8.30 (s, 1H, 8-H), 8.18 (s, 1H, 2-H), 7.50–7.29 (m, 5H, Ar-H), 6.00 (s, 2H, -NH_2_), 5.55 (s, 1H, Ph-CH), 5.15 (s, 1H, 1'-H), 5.07 (s, 1H, 2'-H), 4.64− 4.52 (m, 1H, 5'-H), 4.46 (dd, *J* = 9.9, 4.7 Hz, 1H, 6'-He), 4.33 (brs, 1H, 3'-H), 3.86 (t, *J* = 9.9 Hz, 1H, 6'-Ha), 3.73 (d, *J* = 8.3 Hz, 1H, 4'-H), 3.54 (s, 3H, OMe). ^13^C-NMR (75 MHz, CDCl_3_): δ 155.60 (C-6); 153.44 (C-2); 149.77 (C-4); 138.45 (C-8); 136.86 (C-5); 129.28, 128.26, 126.15, 118.89 (Ar-C); 102.35 (Ph-C); 99.66 (C-1'); 75.87 (C-5'); 69.14 (C-3'); 67.03 (C-4'); 58.52 (C-6'); 57.18 (OMe); 56.13 (C-2'). HRMS calcd. for C_19_H_22_N_5_O_5_^+^ [M+H]^+^ 400.1615, found 400.1613.

### 3.8. 4',6'-O-Benzylidene-1',3'-di-O-methyl-2'-deoxy-2'-(adenin-9-yl)-d-altropyranoside (**8**)

The adenine analog **7** (2.36 g, 5.9 mmol) was co-evaporated with dry DMF (40 mL).The foam was dissolved in dry DMF (70 mL), cooled to −78 °C and NaH (330 mg, 8.28 mmol) was added and the mixture was stirred at −78 °C for 30 min under argon. Subsequently, methyl iodide (0.590 mL, 9.46 mmol) was dissolved in dry DCM (20 mL) and the solution was added drop wise over 30 min to the reaction mixture at the same temperature. Stirring was continued for another 4.5 h at −78 °C and at −30 °C for an additional 1 h before quenching of the reaction with MeOH (10 mL). The solution was warmed to RT and concentrated to dryness under vacuum. The residue was dissolved in ethyl acetate (150 mL) and washed with aqueous saturated NaHCO_3_ (130 mL). The organic layer was then washed with brine (130 mL), and the combined aqueous layers were again extracted with ethyl acetate (2 × 150 mL). The combined organic layers were dried over Na_2_SO_4_, filtered, and concentrated. Purification by silica column chromatography (0%–3% MeOH/DCM) afforded the methylated nucleoside **8** (1.84 g, 75.4%). ^1^H-NMR (500 MHz, CDCl_3_): δ 8.39 (s, 1H, 8-H), 8.20 (s, 1H, 2-H), 7.47–7.40 (m, 2H, Ar-H), 7.37–7.31 (m, 3H, Ar-H), 5.92 (s, 2H, -NH_2_), 5.49 (s, 1H, Ph-CH), 5.15 (d, *J* = 2.5 Hz, 1H, 1'-H), 5.07 (s, 1H, 2'-H), 4.54 (td, *J* = 10.1, 5.3 Hz, 1H, 5'-H), 4.44 (dd, *J* = 10.5, 5.3 Hz, 1H, 6-He), 3.88 (t, *J* = 2.6 Hz, 1H, 3'-H), 3.83 (t, *J* = 10.5 Hz, 1H, 6'-Ha), 3.79 (dd, *J* = 9.8, 2.5 Hz, 1H, 4'-H), 3.71 (s, 3H, OMe), 3.52 (s, 3H, OMe). ^13^C-NMR (125 MHz, CDCl_3_): δ 155.57 (C-6); 153.54 (C-2); 149.99 (C-4); 138.56 (C-8); 137.11, 129.15, 128.26, 126.16 (Ar-C); 119.10 (C-5); 102.48 (Ph-C); 99.47 (C-1'); 76.25 (C-5'); 76.19 (C-3'); 69.26 (C-4'); 60.06 (C-6'); 58.81 (OMe); 56.06 (OMe); 55.05 (C-2'). HRMS calcd. for C_20_H_24_N_5_O_5_^+^ [M+H]^+^ 414.1772, found 414.1767.

### 3.9. 4',6'-O-Benzylidene-1',3'-di-O-methyl-2'-deoxy-2'-(N^6^-benzoyladenin-9-yl)-d-altropyranoside (**9**)

The obtained analog **8** (1.59 g, 3.85 mmol) was co-evaporated with dry pyridine (2 mL), dissolved in dry pyridine (16 mL), and cooled at 0 °C. Benzoyl chloride (1.34 mL, 11.54 mmol) was added and the solution was allowed to come to RT. The solution was stirred 3 h at RT. The mixture was cooled to 0 °C and water (4 mL) was added. Then, aqueous ammonia 25% (8 mL) was added and the solution was stirred 30 min at RT. The volatiles were removed under reduced pressure and co-evaporated, each time with toluene (3 × 25 mL). The residue was adsorbed on silica by co-evaporation and purified by silica column chromatography (elution with 2% MeOH/DCM) affording the protected nucleoside **9** (1.70 g, 83% yield) as a white foam. ^1^H-NMR (500 MHz, CDCl_3_): δ 9.43 (s, 1H, NH), 8.78 (s, 1H, 8-H), 8.43 (s, 1H, 2-H), 8.05 (d, *J* = 7.5 Hz, 2H, H-Bz), 7.66–7.29 (m, 8H, Bz-H, Ar-H), 5.49 (s, 1H, Ph-CH), 5.22 (d, *J* = 2.1 Hz, 1H, 1'-H), 5.09 (s, 1H, 2'-H), 4.55 (td, *J* = 10.0, 5.3 Hz, 1H, 5'-H), 4.44 (dd, *J* = 10.4, 5.2 Hz, 1H, 6'-He), 3.88 (brs, 1H, 3'-H), 3.85–3.76 (m, 2H, ,6'-Ha, 4'-H), 3.71 (s, 3H, OMe), 3.52 (s, 3H, OMe). ^13^C-NMR (125 MHz, CDCl_3_): δ 164.86 (C=O); 152.77 (C-6); 152.06 (C-2); 149.78 (C-4); 141.06 (C-8); 137.01, 133.35, 132.75, 129.09, 128.72, 128.19, 128.00, 126.11 (Ar-C, Bz-C); 122.75 (C-5); 102.43 (Ph-C); 99.19 (C-1'); 76.11 (C-5'); 76.08 (C-3'); 69.16 (C-4'); 59.98 (C-6'); 58.83 (OMe); 56.01 (OMe); 55.13 (C-2'). HRMS calcd. for C_27_H_28_N_5_O_6_^+^ [M+H]^+^ 518.2034, found 518.2031.

### 3.10. 4',6'-O-Benzylidene-1'-O-methyl-2'-deoxy-2'-(2-amino-6-(2-trimethylsilyl-ethoxy)purin-9-yl)-d-altropyranoside (**11**)

A mixture of 4.5 g (15.4 mmol) of **10** and 0.266 g (33.5 mmol) of lithium hydride in 60 mL of dry DMF was stirred under nitrogen at 90 °C for 2 h. After addition of 2.6 g (9.8 mmol) of the epoxide **2** dissolved in 20 mL of dry DMF, stirring was continued for 18 h at 130 °C. The reaction mixture was cooled and evaporated to dryness. The residue was dissolved in ethyl acetate (100 mL) and washed with brine (100 mL). A small part of the base precipitated during extraction and was filtered off and kept for recycling. The aqueous layer was again extracted with EtOAc (3 × 50 mL). The combined organic layers were dried over Na_2_SO_4_, filtered, concentrated under *vacuo*, and purification by silica gel column chromatography (elution with 3% MeOH in DCM) afforded **11** (2.11 g, 42%) while recovering a large point of the starting **10** (1.5 g, 26%). ^1^H-NMR (300 MHz, CDCl_3_): δ 8.03 (s, 1H, 8-H), 7.39 (m, 5H, Ar-H), 5.55 (s, 1H, Ph-CH), 5.27 (d, *J* = 1.8 Hz, 1H, 1'-H), 5.04 (s, 1H, 2'-H), 4.83–4.60 (m, 3H, -NH_2_, 5'-H), 4.59–4.38 (m, 3H, 6'-He, 6'-Ha, 3'-H), 4.36–4.29 (m, 1H, 4'-H), 3.89–3.73 (m, 2H, OCH_2_-), 3.55 (s, 3H, OMe), 1.21–1.10 (m, 9H, -SiC_3_H_9_). ^13^C-NMR (75 MHz, CDCl_3_): δ 161.74 (C-6); 159.43 (C-2); 152.79 (C-4); 137.10 (C-8); 136.53 (Ar-C_i_), 129.53 (Ar-C_p_), 128.34 (Ar-C_m_), 126.46 (Ar-C_o_); 115.06 (C-5); 102.58 (Ph-C); 100.05 (C-1'); 76.14 (C-4'); 69.25 (C-6'); 66.10 (C-5'); 65.09 (C-3'); 58.63 (OCH_2_-); 57.19 (OMe); 56.40 (C-2'); 17.49 (CH_2_Si); −1.46 (-SiC_3_H_9_).

### 3.11. 4',6'-O-Benzylidene-1',3'-di-O-methyl-2'-deoxy-2'-(2-amino-6-(2-trimethylsilylethoxy)-purin-9-yl)-d-altropyranoside (**12**)

The obtained foam **11** (2.11 g, 4.1 mmol) was dried overnight under vacuum. The foam was dissolved in dry DMF (42 mL), cooled to −30 °C and NaH (1.3 g, 32 mmol) was added and the mixture was stirred at −30 °C for 1 h under argon. Subsequently, methyl iodide (1 mL, 16 mmol) was dissolved in dry DCM (10.5 mL) and the solution was added drop wise over 30 min to the reaction mixture at −30 °C which was stirred further at −30 °C for 1 h. The reaction was finally running 3 h more at −30 °C to −20 °C before quenching with 10 mL of MeOH for 20 min. The solution was concentrated and dissolved in DCM (50 mL) and washed with brine (50 mL). The aqueous layer was again extracted with DCM (3 × 50 mL). The combined organic layers were dried over Na_2_SO_4_, filtered and concentrated under *vacuo*, and purification by silica gel column chromatography (elution with 2% MeOH in DCM) afforded methylated nucleoside **12** (2.03 g, 94%) as a white foam. ^1^H-NMR (500 MHz, CDCl_3_): δ 7.98 (s, 1H, 8-H), 7.49–7.40 (m, 1H, 2H, Ar-H), 7.38–7.30 (m, 3H, Ar-H), 5.47 (s, 1H, Ph-CH), 5.01 (s, 1H, 1'-H), 4.98–4.92 (m, 1H, 5'-H), 4.66–4.55 (m, 1H, 3'-H), 4.53–4.46 (m, 1H, 4'-H), 4.41 (dd, *J* = 10.4, 5.3 Hz, 1H, 6'-Ha), 1.26 (t, *J* = 12.1 Hz, 1H). ^13^C-NMR (126 MHz, CDCl_3_): δ 161.59 (C-6); 159.61 (C-2); 153.62 (C-4); 137.21 (C-8); 136.93 (Ar-C_i_); 129.07 (Ar-C_p_); 128.21 (Ar-C_m_); 126.16 (Ar-C_o_); 115.15 (C-5); 102.42 (Ph-C); 99.45 (C-1'); 76.26 (C-4'); 76.06 (C-6'); 69.26 (C-5'); 65.04 (C-3'); 59.98 (OCH_2_-); 58.73 (OMe); 55.97 (OMe); 54.82 (C-2'); 17.54 (CH_2_Si); −1.41 (3C, Si(CH_3_)_3_). HRMS calcd. For C_25_H_36_N_5_O_6_Si^+^ [M+H]^+^ 530.2429, found 530.2430.

### 3.12. 4',6'-O-Benzylidene-1',3'-di-O-methyl-2'-deoxy-2'-(guanin-9-yl)-d-altropyranoside (**13**)

A 1 M solution of tetrabutylammonium fluoride in dry THF (15.10 mL) was added to the guanine derivative **12** (2.0 g , 3.77 mmol) and the mixture was stirred at room temperature under N_2_ for 2 h after which water (15 mL) was added. The pH was adjusted to 5 with acetic acid and the mixture was evaporated. The residue was purified by silica gel column chromatography (elution with 10% MeOH in DCM) affording **13** (1.25 g, 75%). ^1^H-NMR (500 MHz, CDCl_3_): δ 11.99 (s, 1H, NH), 7.86 (s, 1H, 8-H), 7.42 (m, 2H, Ar-H), 7.31 (m, 3H, Ar-H), 6.73 (s, H, 2H, 2-NH_2_), 5.49 (s, 1H, Ph-CH), 5.07 (s, 1H, 1'-H), 4.81 (s, 1H, 2'-H), 4.50–4.42 (m, 1H, 5'-H), 4.37 (dd, *J* = 10.1, 5.0 Hz, 1H, 6'-He), 3.78 (s, 3H, 3'-H, 6-Ha, 4'-H), 3.58 (s, 3H, OMe), 3.53 (s, 3H, OMe). ^13^C-NMR (125 MHz, CDCl_3_): δ 159.03 (C-6); 154.05 (C-2); 151.58 (C-4); 137.20 (Ar-C_i_); 135.42 (C-8); 129.08, 128.22, 126.19 (Ar-C_m+o+p_); 116.24 (C-5); 102.40 (Ph-C); 99.23 (C-1'); 76.11 (C-4'); 76.04 (C-6'); 69.19 (C-5'); 59.76 (C-4'); 58.68 (OMe); 55.98 (OMe); 54.79 (C-2'). HRMS calcd. For C_20_H_24_N_5_O_6_^+^ [M+H]^+^ 430.1721, found 430.1711.

### 3.13. 4',6'-O-Benzylidene-1',3'-di-O-methyl-2'-deoxy-2'-(N^2^-(dimethylamino)methylene-guanin-9-yl))-d-altropyranoside (**14**)

An amount of **13** (0.66 g, 1.54 mmol) was co evaporated three times with pyridine, dissolved in 30 mL of dry MeOH (20 mL) and *N,N*-dimethylformamide diethyl acetal (0.824 mL, 6.16 mmol) was added. The mixture was stirred at reflux for 2 h under argon, after which it was evaporated and co-evaporated with toluene (3 × 30 mL). The residue was purified by silica gel column chromatography (2%–5% MeOH/dichloromethane) affording the analog **14** (0.634 g, 85%). ^1^H-NMR (500 MHz, CDCl_3_): δ 9.78 (s, 1H, N^1^-H), 8.65 (s, 1H, N=CH-N), 7.97 (s, 1H, 8-H), 7.45 (m, 2H, Ar-H), 7.40–7.30 (m, 3H, Ar-H), 5.51 (s, 1H, Ph-CH), 5.02 (s, 1H, 2'-H), 4.99 (d, *J* = 2.2 Hz, 1H, 1'-H), 4.50 (td, *J* = 10.4, 5.3 Hz, 1H, 5'-H), 4.41 (dd, *J* = 10.4, 5.3 Hz, 1H, 6-He), 3.90–3.80 (m, 3H, 3'-H, 6-Ha, 4'-H), 3.68 (s, 3H, OMe), 3.50 (s, 3H, OMe), 3.18 (s, 3H, NMe), 3.15 (s, 3H, NMe). ^13^C-NMR (125 MHz, CDCl_3_): δ 158.12 (N=CH-N); 157.97 (C-6); 157.04 (C-2); 150.18 (C-4); 137.20 (C-8); 136.08, 129.05, 128.19, 126.14 (Ar-C); 119.85 (C-4); 102.39 (Ph-CH); 99.31 (C-1'); 76.47 (C-4'); 76.12 (C-6'); 69.17 (C-3'); 60.14 (C-5'); 58.67 (OMe); 55.89 (OMe); 55.13 (C-2'); 41.44 (NMe); 35.23 (NMe). HRMS calcd. for C_23_H_29_N_6_O_6_^+^ [M+H]^+^ 485.2143, found 485.2145.

### 3.14. 1',3'-Di-O-methyl-2'-deoxy-2'-(uracil-1-yl)-d-altropyranoside (**15a**)

Compound **4** (0.9 g, 2.31 mmol) was dissolved in 60 mL of AcOH-H_2_O (3:1) at rt. The reaction mixture was slowly heated at 45 °C and reaction progress was monitored using TLC. After 12 h, the mixture was concentrated and co evaporated with toluene (30 mL). The crude residue was purification by flash silica gel column chromatography (elution with 5% MeOH in DCM) afforded compound **15a** (0.66 g, 94%). ^1^H-NMR (600 MHz, DMSO-*d*_6_): δ 11.15 (s, 1H, NH), 7.71 (s, 1H, 6-H), 5.56 (d, *J* = 5.5 Hz, 1H, 5-H), 4.93 (d, *J* = 3.9 Hz, 1H, 1'-H), 4.88 (t, *J* = 5.0 Hz, 1H, 2'-H), 4.84–4.60 (m, 1H, 3'-H), 4.03 (dd, *J* = 9.8, 5.5 Hz, 1H, 5'-H), 3.77 (q, *J* = 5.5 Hz, 1H, 4'-H), 3.65–3.53 (m, 2H, 6'-H), 3.26, (s, 3H, OMe), 3.22 (s, 3H, OMe). ^13^C-NMR (150 MHz, DMSO-*d*_6_): δ 170.83 (C-4); 163.61 (C-2); 151.66 (C-6); 101.62 (C-5); 99.07 (C-1'); 77.52 (C-5'); 76.08 (C-3); 63.67 (C-4'); 61.12 (C-6'); 60.23 (OMe); 56.65 (OMe); 55.39 (C-2'). HRMS calcd. For C_12_H_19_N_2_O_7_^+^ [M+H]^+^ 325.1006, found 325.1005.

### 3.15. 1',3'-Di-O-methyl-2'-deoxy-2'-(N^6^-benzoylcytosin-1-yl)-d-altropyranoside (**15b**)

Compound **6** (0.36 g, 0.73 mmol) was dissolved in 24.1 mL of AcOH:H_2_O (3:1) at rt. The reaction mixture was slowly heated at 45 °C and further treated as per the synthesis of **15a** affording the nucleoside analog 1**5b** (0.28 g, 50%). ^1^H-NMR (300 MHz, CD_3_OD): 8.23–7.94 (m, 3H, 6-H, Bz-H), 7.68–7.51 (m, 4H, 5-H, Bz-H), 5.26 (brs, 1H, 1'-H), 4.87–4.81 (m, 1H, 2'-H), 4.23–4.09 (m, 1H, 5'-H), 4.03 (q, 1H, *J* = 5.5 Hz, 4'-H), 3.80–3.66 (m, 2H, 3'-H, 6'-He), 3.30 (s, 6H, 2OMe), 3.26–3.20 (m, 1H, 6'-Ha). ^13^C-NMR (150 MHz, CD_3_OD): 169.07 (PhCONH), 164.69 (C-4), 158.61 (C-2), 134.65 (C-6), 134.13, 130.47, 129.84, 129.59, 129.19 (Bz-C), 100.35 (C-5), 98.59 (C-1'), 64.94 (C-3'), 64.74 (C-5'), 63.46 (C-4'), 62.47 (C-6'), 57.75 (OMe), 56.05 (OMe), 49.85 (C-2'). HRMS calcd. for C_19_H_24_N_3_O_7_^+^ [M+H]^+^ 406.1609, found 406.1608.

### 3.16. 1',3'-Di-O-methyl-2'-deoxy-2'-(N^6^-benzoyladenin-9-yl)-d-altropyranoside (**15c**)

Following the procedure for **15a**, compound **9** (1.65 g, 3.19 mmol) was treated 82.85 mL of AcOH:H_2_O (3:1) at rt. The reaction mixture was slowly heated at 45 °C and reaction progress was monitored using TLC. After 12 h, the mixture was concentrated and co evaporated with toluene, and after purification afforded (0.78 g, 57%) of **15c**.

^1^H-NMR (300 MHz, CD_3_OD): δ 8.76 (s, 1H, 8-H), 8.52 (s, 1H, 2-H), 8.13 (d, *J* = 7.4 Hz, 2H, Bz-H), 7.77–7.53 (m, 3H, Bz-H), 5.39 (d, *J* = 5.5 Hz, 1H, 1'-H), 4.88–4.82 (m, 1H, 2'-H), 4.31 (dd, *J* = 8.8, 3.7 Hz, 1H, 5'-H), 4.26–4.21 (m, 1H, 3'-H), 4.13 (q, *J* = 5.5 Hz, 1H, 4'-H), 4.00–3.9 (m, 2H, 6'-H), 3.42 (s, 3H, OMe), 3.36 (s, 3H, OMe). ^13^C-NMR (75 MHz, DMSO-*d*_6_): δ 165.20 (C=O); 152.25 (C-6); 150.77 (C-2); 149.68 (C-4); 143.85 (C-8); 132.92 (C-5); 131.89, 127.95, 127.91, 125.07 (Bz-C); 97.91 (C-1'); 77.10 (C-5'); 75.44 (C-3'); 62.37 (C-4'); 59.78 (C-6'); 55.75 (OMe); 55.53 (OMe); 54.53 (C-2'). HRMS calcd. for C_20_H_24_N_5_O_6_^+^ [M+H]^+^ 430.1721, found 430.1716.

### 3.17. 1',3'-Di-O-methyl-2'-deoxy-2'-(N^2^-(dimethylamino)methylene-guanin-9-yl))-d-altro-pyranoside (**15d**)

Compound **14** (0.58 g, 1.2 mmol) was dissolved in 30 mL of AcOH:H_2_O (3:1) at rt. The reaction mixture was slowly heated at 45 °C and reaction progress was monitored using TLC. After 12 h, the mixture was concentrated and co-evaporated with toluene. The crude residue was purified by flash silica gel column chromatography (elution with 10% MeOH in DCM) afforded compound **15d** (0.4 g, 81%). ^1^H-NMR (500 MHz, CD_3_OD): δ 8.65 (s, 1H, N=CH-N), 7.85 (s, 1H, 8-H), 5.21 (d, *J* = 5.3 Hz, 1H, 2'-H), 4.64–4.57 (m, 1H, 1'-H), 4.10 (m, 2H, 4'-H, 3'-H), 4.00 (dd, *J* = 8.8, 4.1 Hz, 1H, 5'-H), 3.84 (d, *J* = 4.6 Hz, 2H, 6'-H), 3.33 (s, 3H, OMe), 3.30 (s, 3H, OMe), 3.16 (s, 3H, OMe), 3.07 (s, 3H, OMe). ^13^C-NMR (125 MHz, CD_3_OD): δ 160.31 (N=C-N); 159.83 (C-6); 158.79 (C-2); 152.31 (C-4); 140.37 (C-8); 120.48 (C-5); 100.37 (C-1'); 78.41 (C-5'); 76.47 (C-3'); 65.24 (C-4'); 62.66 (C-6'); 57.94 (OMe); 57.74 (OMe); 56.17 (C-2'); 41.53 and 35.34 (NMe_2_). HRMS calcd. for C_16_H_25_N_6_O_6_^+^ [M+H]^+^ 397.1830, found 397.1827.

### 3.18. 6'-O-Dimethoxytrityl-1',3'-di-O-methyl-2'-deoxy-2'-(uracil-1-yl)-d-altropyranoside (**16a**)

To a solution of the nucleoside **15a** (0.4 g, 1.32 mmol) in anhydrous pyridine (10 mL) and under argon atmosphere, dimethoxytrityl chloride (0.49 g, 1.45 mmol) was added under stirring on an ice bath. After stirring at room temperature for 3 h, 5% aqueous NaHCO_3_ solution (1 mL) was added, the reaction solvent was evaporated, diluted with CH_2_Cl_2_ (50 mL) and washed with 5% aqueous NaHCO_3_ (2 × 30 mL). The aqueous layers were back extracted once with 30 mL CH_2_Cl_2_. The combined organic layer was dried (Na_2_SO_4_), evaporated under reduced pressure and the crude residue was purified by flash chromatography (CH_2_Cl_2_/MeOH 96:4) affording the corresponding dimethoxytritylated nucleoside **16a** (0.745 g, 93% yield) as a white foam. ^1^H-NMR (125 MHz, CDCl_3_): δ 9.31 (brs, 1H, NH), 7.48–7.43 (m, 2H, 6-H, Ar-H), 7.37–7.33 (m, 4H, Ar-H), 7.31–7.26 (m, 2H, Ar-H), 7.24–7.19 (m, 1H, Ar-H), 6.85–6.82 (m, 4H, Ar-H), 5.69 (dd, *J* = 8.0, 1.6 Hz, 1H, 5-H), 5.30 (brs, 1H, 1'-H), 4.94 (brs, 1H, 2'-H), 4.08–3.95 (m, 2H, 5'-H, 3'-H), 3.79 (s, 3H, OMe), 3.78–3.77 (m, 4H, OMe, 4'-H), 3.50–3.42 (brs, s, 6H, 2OMe), 3.42–3.40 (m, 2H, 6'-H). ^13^C-NMR (125 MHz, CDCl_3_): δ 163.28 (C-4); 158.50 (C-2); 150.68 (C-6); 144.85, 135.84, 130.09, 130.06, 128.10, 127.80, 126.81, 113.11 (Ar-C); 102.35 (C-5); 98.36 (C-1'); 86.20 (C^Tr^-O); 77.14 (C-5'), 76.87 (C-3'); 64.05 (C-4'); 62.88 (C-6'); 57.93 (OMe); 55.69 (OMe); 55.19 (2OMe, C-2'). HRMS calcd. for C_33_H_36_N_2_O_9_Na^+^ [M+Na]^+^ 627.23186, found 627.2311.

### 3.19. 6'-O-Dimethoxytrityl-1',3'-di-O-methyl-2'-deoxy-2'-(N^6^-benzoylcytosin-1-yl)-d-altropyranoside (**16b**)

Compound **16b** (0.346 g, 87% yield) was synthesized from compound 1**5b** (0.228 g, 0.56 mmol) using dimethoxytrityl chloride (0.09 g, 1.29 mmol) in anhydrous pyridine (10 mL) according to the procedure used for the synthesis of compound **16a**. ^1^H-NMR (500 MHz, CDCl_3_) : δ 8.75 (brs, 1H, NH), 7.90 (d, *J* = 7.3 Hz, 2H, 6-H, Ar-H), 7.64–7.45 (m, 6H, Ar-H), 7.40–7.19 (m, 8H, 5-H, Ar-H), 6.86 (d, *J* = 8.8 Hz, 4H, Ar-H), 5.29 (s, 1H, 1'-H), 5.03 (brs, 1H, 2'-H), 4.11–3.95 (m, 2H, 5'-H, 3'-H), 3.83–3.77 (m, 7H, 2OMe, 4'-H), 3.60–3.37 (m, 8H, 6'-H, 2OMe), 2.50 (s, 1H, 4-OH). ^13^C-NMR (126 MHz, CDCl_3_): δ 166.13 (C=0), 162.13 (C-4), 158.47 (C-4), 144.71 (C-6), 136.02, 133.17, 130.03, 129.01, 128.20, 127.79, 127.50, 126.80, 113.10 (Ar-C), 98.06 (C-5), 96.85 (C-1'), 86.11 (C^Tr^-O), 77.13 (C-5'), 76.88 (C-3'), 65.62 (C-4'), 62.73 (C-6'), 58.04 (OMe), 55.72 (OMe), 55.18 (C-2, 2OMe). HRMS calcd. For C_40_H_42_N_3_ O_9_^+^ [M+H]^+^ 708.29208, found 708.2909.

### 3.20. 6'-O-Dimethoxytrityl-1',3'-di-O-methyl-2'-deoxy-2'-(N^6^-benzoyladenin-9-yl)-d-altropyranoside (**16c**)

Compound **16c** (0.77 g, 91% yield) was synthesized from compound 1**5c(**0.5 g, 1.16 mmol) using dimethoxytrityl chloride (0.44 g, 1.27 mmol) in anhydrous pyridine (10 mL) according to the procedure used for the synthesis of compound **16a**. ^1^H-NMR (500 MHz, CDCl_3_): δ 9.15 (brs, 1H, 2-H), 8.12 (s, 1H, 8-H),8.03 (d, *J* = 8.0 Hz, 2H, Ar-H), 7.60 (t, *J* = 7.4 Hz, 1H, Ar-H), 7.52–7.49 (m, 3H, Ar-H), 7.40 (d, *J* = 8.8 Hz, 4H, Ar-H), 7.34–7.21 (m, 5H, Ar-H), 6.85 (d, *J* = 8.8 Hz, 3H, Ar-H), 5.29 (t, *J* = 1.1 Hz, 1H, 1'-H), 5.20 (d, *J* = 4.8 Hz, 1H, 2'-H), 4.79–4.68 (m, 1H, 6'-He), 4.27 (q, *J* = 5.5 Hz, 1H, 5'-H), 4.18 (dd, *J* = 7.9, 3.8 Hz, 1H, 4'-H), 4.07 (t, *J* = 4.7 Hz, 1H, 3'-H), 3.79 (s, 6H, OMe), 3.53 – 3.47 (m, 2H, 6'-Ha, OH), 3.41 (s, 3H, OMe), 3.29 (s, 3H, OMe). ^13^C-NMR (125 MHz, CDCl_3_): δ 164.63 (C-6); 158.52 (Ar-C); 152.46 (C-2); 151.78 (C-4); 149.56 (C-8); 144.66, 143.58, 135.84, 133.67, 132.75, 130.05, 128.84, 128.17, 127.85, 126.84, 123.11, 113.16 (Ar-C); 98.31 (C-1'); 86.33 (C^Tr^-O); 76.26 (C-5'); 72.89 (C-3'); 64.68 (C-4'); 62.90 (C-6'); 57.89 (OMe); 56.87 (OMe); 55.94 (OMe); 55.19 (OMe); 53.38 (C-2'). HRMS calcd. for C_41_H_42_N_5_O_8_^+^ [M+H]^+^ 732.30332, found 732.3030.

### 3.21. 6'-O-Dimethoxytrityl-1',3'-di-O-methyl-2'-deoxy-2'-(N^2^-(dimethylamino)methylene-guanin-9-yl))-d-altropyranoside (**16d**)

Compound **16d** (0.51 g, 74% yield) was synthesized from compound 1**5d** (0.39 g, 0.974 mmol) using dimethoxytrityl chloride (0.362 g, 1.07 mmol) in anhydrous pyridine (10 mL) according to the procedure used for the synthesis of compound **16a**. ^1^H-NMR (500 MHz, CDCl_3_): δ 9.20 (s, 1H, NH), 8.61 (d, *J* = 4.2 Hz, 1H, N=CH-N), 8.52 (s, 1H, 8-H), 7.79 (s, 1H, Ar-H), 7.68 (tt, *J* = 7.7, 1.8 Hz, 1H, Ar-H), 7.52–7.48 (m, 2H, Ar-H), 7.41–7.36 (m, 4H, Ar-H), 7.33–7.20 (m, 5H, Ar-H), 6.87–6.82 (m, 4H, Ar-H), 5.29 (s, 1H, 1'-H), 5.09 (d, *J* = 3.1 Hz, 1H, 2'-H), 4.79 (dd, *J* = 6.4, 3.1 Hz, 1H, 3'-H), 4.17–4.11 (m, 1H, 5'-H), 4.01 (brs, 1H, -OH), 3.88 (dd, *J* = 6.4, 4.0 Hz, 1H, 4'-H), 3.52 (dd, *J* = 10.2, 2.9 Hz, 1H, 6-He), 3.46 (s, 3H, OMe), 3.44 (s, 3H, OMe), 3.35 (dd, *J* = 10.2, 5.5 Hz, 1H, 6'-Ha), 3.05 (s, 3H, OMe), 2.93 (s, 3H, OMe). ^13^C-NMR (125 MHz, CDCl_3_): δ 158.50 (N=CH-N); 157.92 (MMTr); 157.78 (C-6); 156.55(C-2); 150.39(C-4); 149.81 (C-8); 144.84 (Ar-C), 137.33, 135.98, 135.87, 130.06, 130.02, 128.10, 127.85, 126.81, 123.70, 120.37, 113.13 (Ar-C); 99.83 (C-1'); 86.17 (C^Tr^-O); 78.04 (C-5'); 71.31 (C-3'); 64.79 (C-4'); 63.87 (C-6'); 58.37 (OMe); 55.65 (OMe); 54.56 (C-2'); 41.12 and 35.12 (-NMe_2_). HRMS calcd. for C_37_H_43_N_6_O_8_^+^ [M+H]^+^ 699.3142, found 699.3121.

### 3.22. General Procedure for Nucleoside Phosphitylation

To a solution of the dimethoxytritylated nucleoside **16a** (0.73 g, 1.2 mmol) in anhydrous CH_2_Cl_2_ (6 mL) at 0 °C and under argon atmosphere, freshly dried diisopropylethylamine (0.063 mL, 3.6 mmol) and 2-cyanoethyl-*N,N*-diisopropylchlorophosphoramidite (0.040 mL, 1.8 mmol) were added. The reaction mixture was stirred at 0 °C for 90 min after which completeness of the reaction was indicated by TLC. Saturated NaHCO_3_ solution (2 mL) was added, the solution was stirred for another10 min and partitioned between CH_2_Cl_2_ (50 mL) and aqueous NaHCO_3_ (30 mL). The organic layer was washed with brine (3 × 30 mL) and the aqueous phases were back extracted with CH_2_Cl_2_ (30 mL). After solvent evaporation, the resulting oil was purified by flash chromatography (hexane/acetone/TEA = 62/36/2). The yellow solid was then dissolved in CH_2_Cl_2_ (2 mL) and precipitated twice in cold hexane (160 mL, −30 °C) to afford the desired corresponding phosphoramidite nucleoside **17a** (0.908 g, 93% yield) as a white powder. The obtained product was dried under vacuum and stored overnight under argon at −20 °C. ^31^P-NMR (CDCl_3_): δ = 150.84. HRMS calcd. for C_42_H_54_N_4_O_10_P_1_^+^ [M+H]^+^ 805.35773, found 805.3557; ^31^P-NMR (CDCl_3_): δ = 150.84.

Compound **17b** (0.34 g, 79% yield) was synthesized from compound **16b** (0.34 g, 0.47 mmol), dry diisopropylethylamine (0.025 mL, 1.42 mmol), 2-cyanoethyl-*N,N*-diisopropylchloro-phosphoramidite (0.016 mL, 0.71 mmol) and anhydrous CH_2_Cl_2_ (10 mL) according to procedure used for the synthesis of compound **17a**. ^31^P-NMR (CDCl_3_): δ = 150.96. HRMS calcd. for C_49_H_59_N_5_O_10_P_1_ [M+H]^+^ 908.39993, found 908.3981.

Compound **17c** (0.83 g, 85% yield) was synthesized from compound **16c** (0.76 g, 1.03 mmol), dry diisopropylethylamine (0.054 mL, 3.09 mmol), 2-cyanoethyl-*N,N*-diisopropylchloro-phosphoramidite (0.034 mL, 1.54 mmol) and anhydrous CH_2_Cl_2_ (10 mL) according to procedure used for the synthesis of compound **17a**. ^31^P-NMR (CDCl_3_): δ = 150.287 and 151.231. HRMS calcd. for C_50_H_59_N_7_O_9_P_1_^+^ [M+H]^+^ 932.41116, found 932.4103.

Compound **17d** (0.42 g, 65% yield) was synthesized from compound **16d** (0.5 g, 0.71 mmol), dry diisopropylethylamine (0.037 mL, 2.13 mmol), 2-cyanoethyl-*N,N*-diisopropylchloro-phosphoramidite (0.025 mL, 1.15 mmol) and anhydrous CH_2_Cl_2_ (10 mL) according to procedure used for the synthesis of compound **17a**. ^31^P-NMR (CDCl_3_): δ = 150.065 and 151.206. HRMS calcd. for C_46_H_60_N_8_O_9_P_1_^+^ [M+H]^+^ 899.42206, found 899.4240.

## 4. Conclusions

A new nucleoside analogue scaffold for incorporation into oligonucleotides was developed and all four monomers with the natural heterocyclic bases have been prepared and evaluated on their hybridization potential with natural DNA and RNA. While it was anticipated that the constraint imposed by the 6-membered ring structure could afford the entropic advantage as seen with HNA and ANA constructs, an entropic penalty to preserve the ^1^C_4_ conformation required for pairing with RNA annulated the affinity gain which one could expect from a pre-organized structure.
